# 2-(2,6-Dichloro­phen­yl)-*N*-(1,3-thia­zol-2-yl)acetamide

**DOI:** 10.1107/S1600536813006260

**Published:** 2013-03-09

**Authors:** Prakash S. Nayak, B. Narayana, H. S. Yathirajan, Jerry P. Jasinski, Ray J. Butcher

**Affiliations:** a Department of Studies in Chemistry, Mangalore University, Mangalagangotri 574 199, India; b Department of Studies in Chemistry, University of Mysore, Manasagangotri, Mysore 570 006, India; c Department of Chemistry, Keene State College, 229 Main Street, Keene, NH 03435-2001, USA; d Department of Chemistry, Howard University, 525 College Street NW, Washington, DC 20059, USA

## Abstract

In the title compound, C_11_H_8_Cl_2_N_2_OS, the mean plane of the dichloro­phenyl ring is twisted by 79.7 (7)° from that of the thia­zole ring. In the crystal, molecules are linked *via* pairs of N—H⋯N hydrogen bonds, forming inversion dimers which stack along the *a*-axis direction.

## Related literature

For the structural similarity of N-substituted 2-aryl­acetamides to the lateral chain of natural benzyl­penicillin, see: Mijin & Marinkovic (2006 ▶); Mijin *et al.* (2008 ▶). For the coordination abilities of amides, see: Wu *et al.* (2008 ▶, 2010 ▶). For related structures, see: Fun *et al.* (2012*a* ▶,*b* ▶,*c* ▶,*d* ▶,*e* ▶); Butcher *et al.* (2013*a* ▶,*b* ▶). For standard bond lengths, see: Allen *et al.* (1987 ▶).
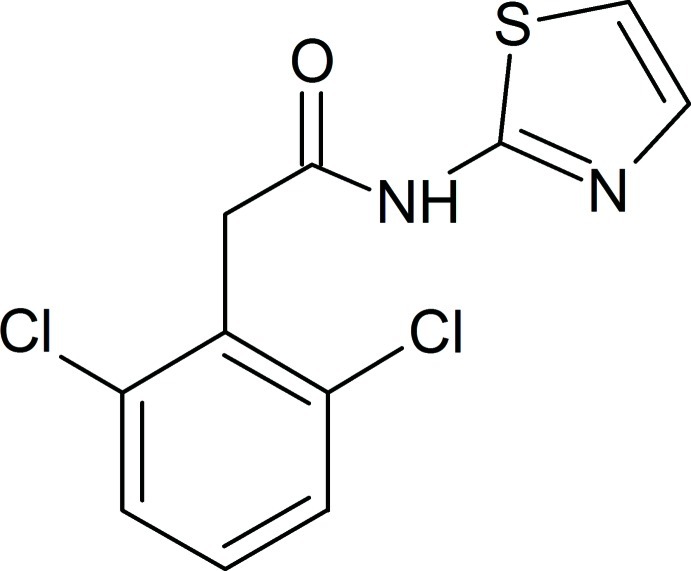


## Experimental

### 

#### Crystal data

C_11_H_8_Cl_2_N_2_OS
*M* *_r_* = 287.15
Triclinic, 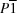

*a* = 7.1278 (4) Å
*b* = 8.4434 (4) Å
*c* = 10.3637 (6) Å
α = 95.341 (4)°
β = 106.140 (5)°
γ = 96.745 (4)°
*V* = 589.80 (6) Å^3^
*Z* = 2
Mo *K*α radiation
μ = 0.71 mm^−1^
*T* = 123 K
0.3 × 0.2 × 0.1 mm

#### Data collection

Agilent Xcalibur (Ruby, Gemini) diffractometer
Absorption correction: multi-scan (*CrysAlis RED*; Agilent, 2012 ▶) *T*_min_ = 0.909, *T*_max_ = 1.000
10674 measured reflections
5881 independent reflections
4481 reflections with *I* > 2σ(*I*)
*R*_int_ = 0.025

#### Refinement

*R*[*F* ^2^ > 2σ(*F* ^2^)] = 0.041
*wR*(*F* ^2^) = 0.096
*S* = 1.05
5881 reflections
159 parameters
H atoms treated by a mixture of independent and constrained refinement
Δρ_max_ = 0.63 e Å^−3^
Δρ_min_ = −0.30 e Å^−3^

### 

Data collection: *CrysAlis PRO* (Agilent, 2012 ▶); cell refinement: *CrysAlis PRO*; data reduction: *CrysAlis PRO*; program(s) used to solve structure: *SHELXS97* (Sheldrick, 2008 ▶); program(s) used to refine structure: *SHELXL97* (Sheldrick, 2008 ▶); molecular graphics: *SHELXTL* (Sheldrick, 2008 ▶); software used to prepare material for publication: *SHELXTL*.

## Supplementary Material

Crystal structure: contains datablock(s) global, I. DOI: 10.1107/S1600536813006260/hg5297sup1.cif

Structure factors: contains datablock(s) I. DOI: 10.1107/S1600536813006260/hg5297Isup2.hkl

Supplementary material file. DOI: 10.1107/S1600536813006260/hg5297Isup3.cml

Additional supplementary materials:  crystallographic information; 3D view; checkCIF report

## Figures and Tables

**Table 1:** Hydrogen-bond geometry (Å, °)

*D*—H⋯*A*	*D*—H	H⋯*A*	*D*⋯*A*	*D*—H⋯*A*
N1—H1*N*⋯N2^i^	0.890 (19)	2.030 (19)	2.9165 (14)	173.9 (17)

Symmetry code: (i) 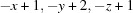.

**Fig. 1.**
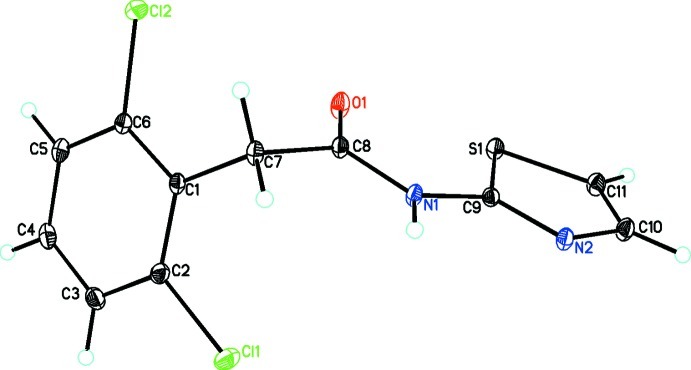
Molecular structure of the title compound showing the atom labeling scheme and 30% probability displacement ellipsoids.

**Fig. 2.**
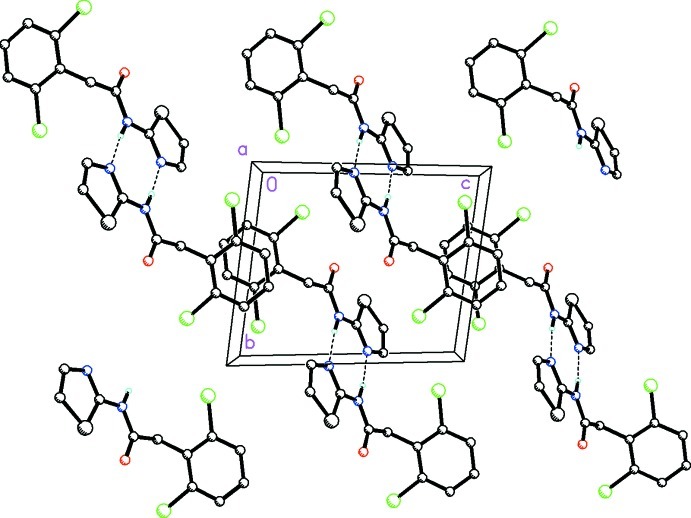
Packing diagram of the title compound viewed along the a axis. Dashed lines indicate N—H···N intermolecular hydrogen bonds which form chains along [100]. H atoms not involved in hydrogen bonding have been removed for clarity.

**Fig. 3.**
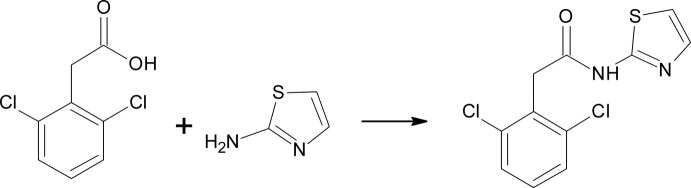
Reaction scheme.

## supplementary crystallographic information

### Comment

N-Substituted 2-arylacetamides are very interesting compounds because of their structural similarity to the lateral chain of natural benzylpenicillin (Mijin *et al.*, 2006, 2008). Amides are also used as ligands due to their excellent coordination abilities (Wu *et al.*, 2008, 2010). Crystal structures of some acetamide derivatives viz., (2,2-diphenyl-N-(1,3-thiazol-2-yl)acetamide, 2-(4-chlorophenyl)-N-(1,3-thiazol-2-yl)acetamide, 2-(naphthalen-1-yl)-N-(1,3-thiazol-2-yl)acetamide, N-(1,3-thiazol-2-yl)-2-(2,4,6-trimethyl phenyl)acetamide, 2-(2-fluorophenyl)-N-(1,3-thiazol-2-yl)acetamide (Fun *et al.*, 2012*a*,*b*,*c*,*d*,*e*), 2-(2,6-dichlorophenyl)-N-(1,5-dimethyl-3-oxo-2-phenyl-2,3-dihydro- 1H-pyrazol-4-yl)acetamide, 2-(2,4-Dichlorophenyl)-N-(1,5-dimethyl-3-oxo- 2-phenyl-2,3-dihydro-1H-pyrazol-4-yl)acetamide (Butcher *et al.*, 2013*a*,*b*) have been reported. In view of the importance of amides, we report herein the crystal structure of the title compound, C_11_H_8_Cl_2_N_2_OS, (I).

In (I), the mean plane of the dichlorophenyl ring is twisted by 79.7 (7)° from that of the thiazol ring (Fig. 1). Bond lengths are in normal ranges (Allen *et al.*, 1987) In the crystal, intermolecular N—H···N hydrogen bonds forming a R2,2(8) graph-set orientation are observed (Table 1) which form infinite 1-D chains along [100] and add to packing stability (Fig. 2).

### Experimental

2,6-Dichlorophenylacetic acid (0.240 g, 1 mmol) and 2-aminothiazole (0.1 g, 1 mmol), 1-ethyl-3-(3-dimethylaminopropyl)-carbodiimide hydrochloride (1.0 g, 0.01 mol) and were dissolved in dichloromethane (20 mL). The mixture was stirred in presence of triethylamine at 273 K for about 3 h. The contents were poured into 100 ml of ice-cold aqueous hydrochloric acid with stirring, which was extracted thrice with dichloromethane (Fig. 3). The organic layer was washed with saturated NaHCO_3_ solution and brine solution, dried and concentrated under reduced pressure to give the title compound (I). Single crystals were grown from methanol and acetone mixture (1:1) by the slow evaporation method (M.P.: 489–491K).

### Refinement

All of the H atoms were placed in their calculated positions and then refined using the riding model with Atom—H lengths of 0.95Å (CH) or 0.99Å (CH_2_). Isotropic displacement parameters for these atoms were set to 1.18-1.23 (CH, CH_2_) times *U*_eq_ of the parent atom.

### Crystal data

**Table:**

C_11_H_8_Cl_2_N_2_OS	*Z* = 2
*M**_r_* = 287.15	*F*(000) = 292
Triclinic, *P*1	*D*_x_ = 1.617 Mg m^−^^3^
Hall symbol: -P 1	Mo *K*α radiation, λ = 0.71073 Å
*a* = 7.1278 (4) Å	Cell parameters from 3814 reflections
*b* = 8.4434 (4) Å	θ = 3.1–37.5°
*c* = 10.3637 (6) Å	µ = 0.71 mm^−^^1^
α = 95.341 (4)°	*T* = 123 K
β = 106.140 (5)°	Prism, colorless
γ = 96.745 (4)°	0.3 × 0.2 × 0.1 mm
*V* = 589.80 (6) Å^3^	

### Data collection

**Table:**

Agilent Xcalibur (Ruby, Gemini) diffractometer	5881 independent reflections
Radiation source: Enhance (Mo) X-ray Source	4481 reflections with *I* > 2σ(*I*)
Graphite monochromator	*R*_int_ = 0.025
Detector resolution: 10.5081 pixels mm^-1^	θ_max_ = 37.6°, θ_min_ = 3.1°
ω scans	*h* = −12→12
Absorption correction: multi-scan (*CrysAlis RED*; Agilent, 2012)	*k* = −13→14
*T*_min_ = 0.909, *T*_max_ = 1.000	*l* = −17→8
10674 measured reflections	

### Refinement

**Table:**

Refinement on *F*^2^	Primary atom site location: structure-invariant direct methods
Least-squares matrix: full	Secondary atom site location: difference Fourier map
*R*[*F*^2^ > 2σ(*F*^2^)] = 0.041	Hydrogen site location: inferred from neighbouring sites
*wR*(*F*^2^) = 0.096	H atoms treated by a mixture of independent and constrained refinement
*S* = 1.05	*w* = 1/[σ^2^(*F*_o_^2^) + (0.0399*P*)^2^ + 0.0356*P*] where *P* = (*F*_o_^2^ + 2*F*_c_^2^)/3
5881 reflections	(Δ/σ)_max_ = 0.001
159 parameters	Δρ_max_ = 0.63 e Å^−^^3^
0 restraints	Δρ_min_ = −0.30 e Å^−^^3^

### Special details

**Table:**

Geometry. All esds (except the esd in the dihedral angle between two l.s. planes) are estimated using the full covariance matrix. The cell esds are taken into account individually in the estimation of esds in distances, angles and torsion angles; correlations between esds in cell parameters are only used when they are defined by crystal symmetry. An approximate (isotropic) treatment of cell esds is used for estimating esds involving l.s. planes.
Refinement. Refinement of *F*^2^ against ALL reflections. The weighted *R*-factor *wR* and goodness of fit *S* are based on *F*^2^, conventional *R*-factors *R* are based on *F*, with *F* set to zero for negative *F*^2^. The threshold expression of *F*^2^ > σ(*F*^2^) is used only for calculating *R*-factors(gt) *etc*. and is not relevant to the choice of reflections for refinement. *R*-factors based on *F*^2^ are statistically about twice as large as those based on *F*, and *R*- factors based on ALL data will be even larger.

### Fractional atomic coordinates and isotropic or equivalent isotropic displacement parameters (Å^2^)

**Table:**

	*x*	*y*	*z*	*U*_iso_*/*U*_eq_	
S1	0.07341 (5)	0.70227 (3)	0.53736 (3)	0.01622 (7)	
Cl1	0.33730 (6)	0.82751 (4)	0.07966 (4)	0.02553 (8)	
Cl2	0.41255 (5)	0.22491 (3)	0.21194 (3)	0.02086 (7)	
O1	0.23281 (15)	0.51841 (10)	0.38090 (10)	0.02131 (19)	
N1	0.38161 (16)	0.77833 (11)	0.43229 (10)	0.01460 (18)	
H1N	0.472 (3)	0.854 (2)	0.4214 (18)	0.033 (5)*	
N2	0.30837 (16)	0.97170 (11)	0.58210 (10)	0.01568 (18)	
C1	0.36862 (17)	0.52292 (13)	0.13841 (11)	0.01338 (19)	
C2	0.29093 (19)	0.61876 (14)	0.03922 (13)	0.0166 (2)	
C3	0.1754 (2)	0.55517 (16)	−0.09107 (13)	0.0203 (2)	
H3A	0.1266	0.6242	−0.1559	0.024*	
C4	0.1326 (2)	0.38945 (16)	−0.12497 (13)	0.0203 (2)	
H4A	0.0532	0.3444	−0.2135	0.024*	
C5	0.20517 (19)	0.28927 (14)	−0.03037 (13)	0.0183 (2)	
H5A	0.1757	0.1757	−0.0536	0.022*	
C6	0.32131 (18)	0.35632 (13)	0.09860 (12)	0.0146 (2)	
C7	0.49054 (18)	0.59521 (14)	0.28024 (12)	0.0154 (2)	
H7A	0.5705	0.6980	0.2768	0.019*	
H7B	0.5820	0.5211	0.3199	0.019*	
C8	0.35693 (18)	0.62512 (13)	0.36893 (12)	0.0147 (2)	
C9	0.26992 (17)	0.82641 (13)	0.51500 (11)	0.01340 (19)	
C10	0.1753 (2)	0.98851 (14)	0.65603 (13)	0.0184 (2)	
H10A	0.1797	1.0856	0.7118	0.022*	
C11	0.0385 (2)	0.85772 (14)	0.64385 (13)	0.0184 (2)	
H11B	−0.0622	0.8526	0.6878	0.022*	

### Atomic displacement parameters (Å^2^)

**Table:**

	*U*^11^	*U*^22^	*U*^33^	*U*^12^	*U*^13^	*U*^23^
S1	0.01826 (14)	0.01396 (12)	0.01733 (13)	−0.00207 (9)	0.00928 (11)	−0.00039 (10)
Cl1	0.03479 (19)	0.01430 (12)	0.03140 (17)	0.00564 (11)	0.01460 (14)	0.00504 (11)
Cl2	0.02711 (16)	0.01518 (12)	0.02042 (14)	0.00524 (10)	0.00606 (12)	0.00315 (10)
O1	0.0265 (5)	0.0151 (4)	0.0240 (5)	−0.0047 (3)	0.0152 (4)	−0.0035 (3)
N1	0.0184 (5)	0.0107 (4)	0.0159 (4)	−0.0013 (3)	0.0095 (4)	−0.0019 (3)
N2	0.0189 (5)	0.0130 (4)	0.0161 (4)	0.0000 (3)	0.0088 (4)	−0.0014 (3)
C1	0.0147 (5)	0.0136 (4)	0.0131 (5)	0.0019 (4)	0.0070 (4)	−0.0008 (4)
C2	0.0195 (5)	0.0148 (5)	0.0193 (5)	0.0043 (4)	0.0108 (4)	0.0030 (4)
C3	0.0222 (6)	0.0264 (6)	0.0160 (5)	0.0085 (5)	0.0089 (5)	0.0054 (5)
C4	0.0175 (6)	0.0291 (6)	0.0136 (5)	0.0053 (5)	0.0045 (4)	−0.0025 (4)
C5	0.0182 (5)	0.0178 (5)	0.0176 (5)	0.0020 (4)	0.0055 (4)	−0.0044 (4)
C6	0.0157 (5)	0.0141 (4)	0.0145 (5)	0.0027 (4)	0.0058 (4)	−0.0002 (4)
C7	0.0160 (5)	0.0153 (5)	0.0148 (5)	−0.0007 (4)	0.0068 (4)	−0.0029 (4)
C8	0.0171 (5)	0.0136 (4)	0.0132 (5)	0.0005 (4)	0.0057 (4)	−0.0014 (4)
C9	0.0157 (5)	0.0124 (4)	0.0127 (4)	0.0008 (4)	0.0060 (4)	0.0007 (4)
C10	0.0230 (6)	0.0153 (5)	0.0196 (5)	0.0024 (4)	0.0117 (5)	−0.0009 (4)
C11	0.0200 (6)	0.0192 (5)	0.0190 (5)	0.0018 (4)	0.0119 (5)	0.0001 (4)

### Geometric parameters (Å, º)

**Table:**

S1—C11	1.7212 (13)	C2—C3	1.3901 (18)
S1—C9	1.7306 (11)	C3—C4	1.3867 (19)
Cl1—C2	1.7436 (12)	C3—H3A	0.9500
Cl2—C6	1.7382 (12)	C4—C5	1.3854 (18)
O1—C8	1.2266 (14)	C4—H4A	0.9500
N1—C8	1.3637 (14)	C5—C6	1.3879 (17)
N1—C9	1.3870 (15)	C5—H5A	0.9500
N1—H1N	0.890 (19)	C7—C8	1.5217 (17)
N2—C9	1.3114 (14)	C7—H7A	0.9900
N2—C10	1.3863 (16)	C7—H7B	0.9900
C1—C2	1.4006 (16)	C10—C11	1.3562 (17)
C1—C6	1.4021 (15)	C10—H10A	0.9500
C1—C7	1.5121 (16)	C11—H11B	0.9500
			
C11—S1—C9	88.83 (6)	C5—C6—Cl2	117.37 (9)
C8—N1—C9	123.21 (10)	C1—C6—Cl2	119.94 (9)
C8—N1—H1N	120.7 (12)	C1—C7—C8	110.36 (10)
C9—N1—H1N	116.1 (12)	C1—C7—H7A	109.6
C9—N2—C10	109.52 (10)	C8—C7—H7A	109.6
C2—C1—C6	115.65 (11)	C1—C7—H7B	109.6
C2—C1—C7	121.89 (10)	C8—C7—H7B	109.6
C6—C1—C7	122.43 (10)	H7A—C7—H7B	108.1
C3—C2—C1	122.99 (11)	O1—C8—N1	122.19 (11)
C3—C2—Cl1	117.80 (9)	O1—C8—C7	121.94 (10)
C1—C2—Cl1	119.20 (9)	N1—C8—C7	115.86 (10)
C4—C3—C2	118.98 (11)	N2—C9—N1	121.19 (10)
C4—C3—H3A	120.5	N2—C9—S1	115.54 (9)
C2—C3—H3A	120.5	N1—C9—S1	123.27 (8)
C5—C4—C3	120.31 (12)	C11—C10—N2	115.85 (11)
C5—C4—H4A	119.8	C11—C10—H10A	122.1
C3—C4—H4A	119.8	N2—C10—H10A	122.1
C4—C5—C6	119.38 (11)	C10—C11—S1	110.26 (9)
C4—C5—H5A	120.3	C10—C11—H11B	124.9
C6—C5—H5A	120.3	S1—C11—H11B	124.9
C5—C6—C1	122.69 (11)		
			
C6—C1—C2—C3	−0.48 (18)	C6—C1—C7—C8	−91.89 (13)
C7—C1—C2—C3	−178.78 (11)	C9—N1—C8—O1	0.06 (19)
C6—C1—C2—Cl1	178.78 (9)	C9—N1—C8—C7	179.00 (10)
C7—C1—C2—Cl1	0.48 (16)	C1—C7—C8—O1	53.64 (15)
C1—C2—C3—C4	0.69 (19)	C1—C7—C8—N1	−125.30 (11)
Cl1—C2—C3—C4	−178.58 (10)	C10—N2—C9—N1	179.79 (11)
C2—C3—C4—C5	−0.38 (19)	C10—N2—C9—S1	−0.23 (13)
C3—C4—C5—C6	−0.09 (19)	C8—N1—C9—N2	174.79 (11)
C4—C5—C6—C1	0.30 (19)	C8—N1—C9—S1	−5.18 (17)
C4—C5—C6—Cl2	−178.67 (10)	C11—S1—C9—N2	0.53 (10)
C2—C1—C6—C5	−0.02 (17)	C11—S1—C9—N1	−179.49 (10)
C7—C1—C6—C5	178.27 (11)	C9—N2—C10—C11	−0.31 (16)
C2—C1—C6—Cl2	178.92 (9)	N2—C10—C11—S1	0.70 (15)
C7—C1—C6—Cl2	−2.79 (16)	C9—S1—C11—C10	−0.66 (10)
C2—C1—C7—C8	86.29 (13)		

### Hydrogen-bond geometry (Å, º)

**Table:**

*D*—H···*A*	*D*—H	H···*A*	*D*···*A*	*D*—H···*A*
N1—H1*N*···N2^i^	0.890 (19)	2.030 (19)	2.9165 (14)	173.9 (17)

Symmetry code: (i) −*x*+1, −*y*+2, −*z*+1.
